# Critical role for NLRP3 in necrotic death triggered by *Mycobacterium tuberculosis*

**DOI:** 10.1111/j.1462-5822.2011.01625.x

**Published:** 2011-03-11

**Authors:** Ka-Wing Wong, William R Jacobs Jr

**Affiliations:** 1 Howard Hughes Medical Institute, Department of Microbiology and Immunology, Albert Einstein College of MedicineBronx, NY 10461, USA

## Abstract

Induction of necrotic death in macrophages is a primary virulence determinant of *Mycobacterium tuberculosis*. The ESX-1 secretion system and its substrate ESAT-6 are required for *M. tuberculosis* to induce necrosis, but host factors that mediate the ESAT-6-promoted necrosis remain unknown. Here we report that ESAT-6-promoted necrotic death in THP-1 human macrophages is dependent on the NLRP3 inflammasome, as shown by RNA interference and pharmacological inhibitions. Phagosomes containing ESAT-6-expressing *M. tuberculosis* recruit markers previously associated with damaged phagosomal membrane, such as galectin-3 and ubiquitinated protein aggregates. In addition, ESAT-6 promoted lysosomal permeabilization by *M. tuberculosis*. ESAT-6 mutants defective for ubiquitination were unable to trigger NLRP3 activation and necrotic death. Furthermore, Syk tyrosine kinase, recently implicated in NLRP3 activation during fungal and malarial infections, was necessary for mediating the ESAT-6-promoted necrosis and NLRP3 activation. Our results thus link phagosomal damage and Syk activity to NLRP3-mediated necrotic death triggered by *M. tuberculosis* ESAT-6 during infection.

## Introduction

*Mycobacterium tuberculosis* is responsible for several million deaths annually and causes devastating illness in infected individuals. Tuberculosis is characterized by the presence of caseous necrotic lesions in the lungs, which are mainly composed of cellular corpses that result from necrotic death in macrophages infected by *M. tuberculosis* (Russell *et al*., 2009). Induction of necrosis induction by *M. tuberculosis* is tightly associated with the virulence of *M. tuberculosis* (Hsu *et al*., 2003; Pan *et al*., 2005). Several laboratories, including ours, have identified the ESX-1 secretion system as the primary determinant for inducing necrosis in macrophages (Hsu *et al*., 2003; Lewis *et al*., 2003). Recent advances in understanding how *M. tuberculosis* induces necrosis have focused on addressing how *M. tuberculosis* blocks host responses that are aimed at avoiding or minimizing necrosis (Gan *et al*., 2008; Divangahi *et al*., 2009). However, the mechanism that describes how *M. tuberculosis* ESX-1 triggers necrosis and which host factors mediate the ESX-1-promoted necrosis remains unknown.

NLRP3 is a cytosolic innate immune sensor that detects a variety of intracellular danger signals (Schroder and Tschopp, 2010). NLRP3 initiates a proinflammatory response by forming a macromolecular complex, termed inflammasome, with the adaptor protein ASC. Inflammasome is responsible for the activation of caspase-1, which in turn transforms pro-IL-1beta and pro-IL-18 into their mature forms. NLRP3 has recently been implicated in necrotic death in macrophages triggered by bacterial infections. Two different types of NLRP3-dependent necrosis have been reported. One of these, known as pyroptosis, requires the inflammasome activity of NLRP3 that is mediated by caspase-1 (Bergsbaken *et al*., 2009). The second type, called pyronecrosis, is caspase-1-independent (Ting *et al*., 2008). A common feature of both types of bacterially triggered NLRP3-dependent necrosis is the requirement of a pore-forming activity. ESAT-6, a major substrate of the ESX-1 system, is known to exhibit pore-forming activity and also demonstrates potent NLRP3-activating ability (Hsu *et al*., 2003; Smith *et al*., 2008; Mishra *et al*., 2010). Based on these observations, we hypothesized that NLRP3 activation by ESAT-6 was a key step in promoting necrotic death in macrophages infected by *M. tuberculosis*.

In this study, a role for NLRP3 in *M. tuberculosis* ESAT-6-promoted necrosis in macrophages was shown by using two independent inhibitors of NLRP3, along with siRNA treatment against NLRP3. Because NLRP3 activation by molecules derived from the hosts is commonly associated with destabilization of phagolysosomes, we also analysed the fate of phagosomes containing *M. tuberculosis*. Our analysis supported the notion that *M. tuberculosis* destabilized its phagosome in an ESAT-6-dependent fashion. The significance of these ESAT-6-induced phagosomal effects on NLRP3 activation was assessed through the use of ESAT-6 point mutants that were defective in destabilizing phagosomes. Furthermore, we explored the involvement of Syk kinase activity, which is atypical for bacterial infections, in the NLRP3-dependent events triggered by ESAT-6. Taken together, our data implicated phagosomal damage and Syk activity as two host factors that contributed to the NLRP3-mediated necrotic death triggered by *M. tuberculosis* ESAT-6 during infection.

## Results

### NLRP3 mediates ESAT-6-dependent necrotic death of human macrophages by *M. tuberculosis*

**Figure 1:**
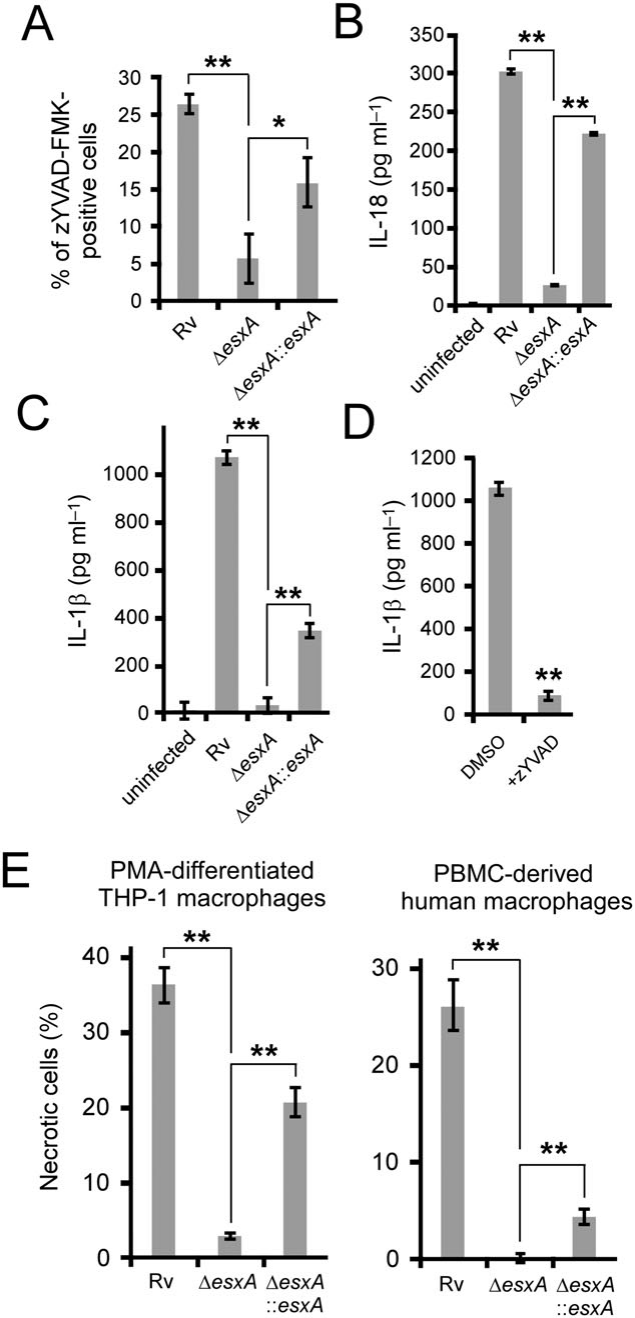
The effect of ESAT-6 on NLRP3 functions during *M. tuberculosis* infections. Differentiated THP-1 macrophages (A–E) or primary human macrophages (E) were infected with the indicated strains of *M. tuberculosis* H37Rv for 2 days. A. The levels of active caspase-1 were determined by staining of active caspase-1 with zYVAD-FMK using fluorescence microscopy. ELISA measurements of IL-18 secretion (B) or IL-1beta (C) were performed on culture supernatants of THP-1 macrophages infected with the indicated strains. D. The effect of 50 µM of the caspase-1 inhibitor, z-YVAD, on IL-1beta secretion from and necrosis of THP-1 macrophages infected with H37Rv. E. The percentages of infected THP-1 macrophages (day 2) or primary human macrophages derived from PBMC (day 3) that became necrotic were determined by fluorescence microscopy. Results are summarized as means ± standard errors. **P < 0.005; *P < 0.05 (relative to DMSO control unless otherwise stated).

**Figure 2:**
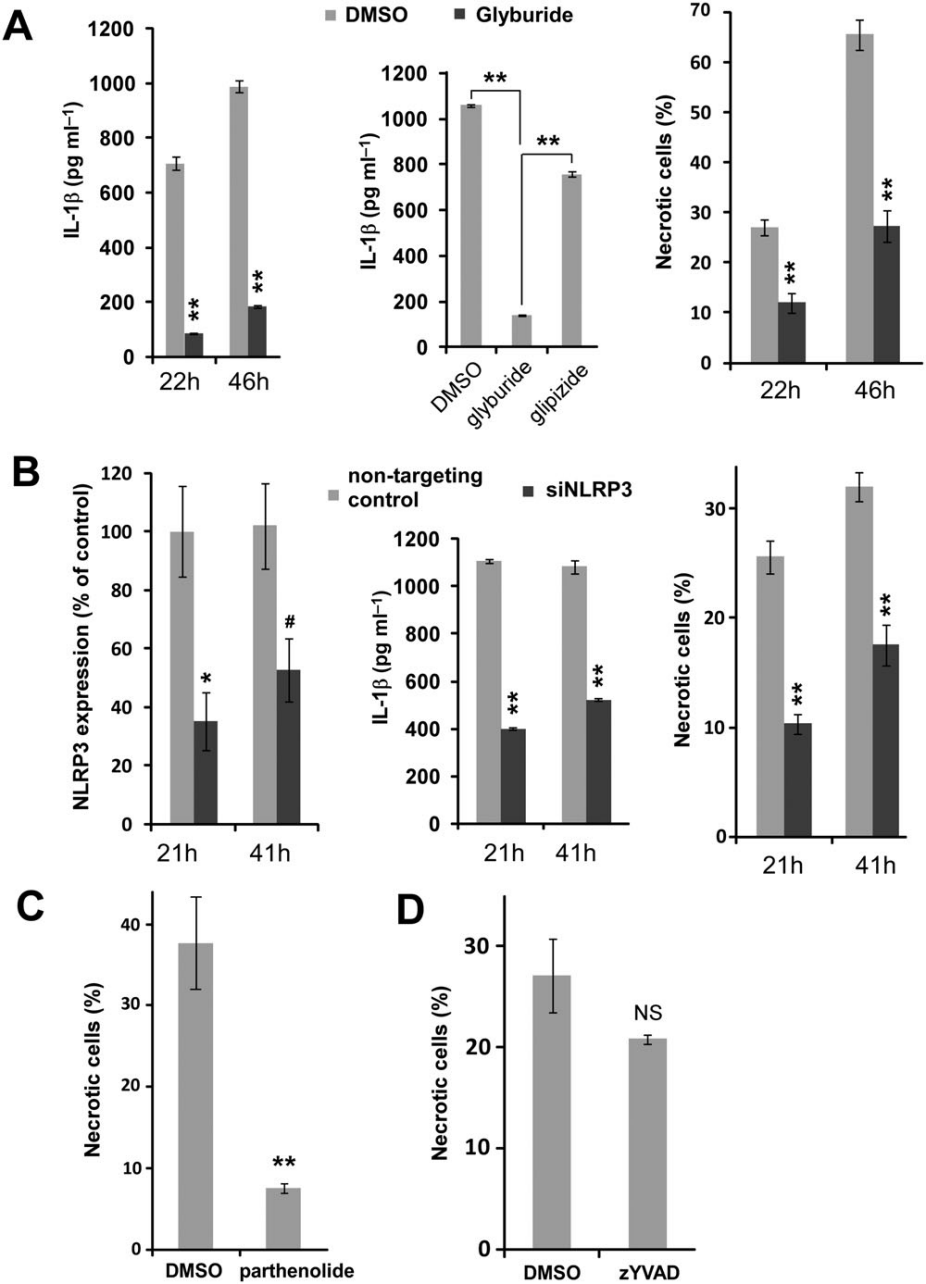
Induction of necrosis in response to *M. tuberculosis* infection is mediated by NLRP3. A. Glyburide treatment. 50 µM glyburide or DMSO as vehicle control was used to pretreat THP-1 macrophages and incubate infected macrophages. IL-1 β levels of culture supernatant and necrosis of infected THP-1 macrophages were analysed at 22 and 46 h after infection. 50 µM glipizide was used as negative control for glyburide. **P < 0.005 (relative to DMSO control unless otherwise stated). B. NLRP3 knockdown by siRNA. THP-1 macrophages were treated with 100 nM siRNA against NLRP3 or non-targeting siRNA as control, as described in *Experimental procedures*. Efficiency of NLRP3 siRNA was determined by real-time PCR as described in *Experimental procedures*. IL-1β levels of culture supernatant and necrosis of infected THP-1 macrophages were analysed at 21 and 41 h post infection. **P < 0.005; *P < 0.05; #*P* = 0.05 (relative to non-targeting siRNA control). C–D. THP-1 macrophages were treated with DMSO, 10 µM parthenolide (C) or 10 µM parthenolide (D). Necrosis levels were measured at 2 days after infection. All results were summarized as means ± standard errors. **P < 0.005; NS: not significant (relative to DMSO control).

*Mycobacterium tuberculosis* ESAT-6 is known to activate NLRP3 (Mishra *et al*., 2010). Because NLRP3 can mediate necrotic death stimulated by intracellular pathogenic bacteria (Ting *et al*., 2008), we hypothesized that the necrotic death stimulated by *M. tuberculosis* was dependent on activation of NLRP3 by ESAT-6. Comparison of the Δ*esxA* strain with the parental strain and complemented strains indicated that the presence of ESAT-6 in *M. tuberculosis* led to upregulations of caspase-1 activation and the caspase-1-dependent secretions of IL-1beta, and IL-18 secretion (Fig. 1A–D), confirming the ESAT-6-dependent activation of NLRP3 by *M. tuberculosis*. Each of these phenotypes was previously known to be mediated by NLRP3 (Schroder and Tschopp, 2010). The role played by ESAT-6 in *M. tuberculosis*-induced necrosis was demonstrated in THP-1 human macrophages (Fig. 1E). Importantly, we were able to confirm this result in primary human macrophages (Fig. 1E). To test the role for NLRP3 in ESAT-6-promoted necrosis, we inactivated NLRP3 function by chemical or genetic inhibition. Compound glyburide, which is known to inhibit NLRP3 function (Lamkanfi *et al*., 2009), blocked IL-1beta production because of *M. tuberculosis* and *M. tuberculosis-*promoted necrosis (Fig. 2A). The specific effect of glyburide was confirmed by showing that glipizide, which is structurally similar to glyburide but does not inhibit NLRP3 (Lamkanfi *et al*., 2009), did not affect the ability of *M. tuberculosis* to induce IL-1beta production (Fig. 2A) or necrosis (data not shown). As a complementary approach, we targeted NLRP3 by siRNA treatment and showed that the siRNA treatment inhibited both IL-1beta secretion and necrosis induced by *M. tuberculosis* (Fig. 2B). Finally, parthenolide, a recently reported NLRP3 inhibitor (Juliana *et al*., 2010), also blocked the level of necrosis stimulated by *M. tuberculosis* (Fig. 2C). NLRP3-dependent necrotic deaths stimulated by pathogenic bacteria typically involve the caspase-1 inflammasome activity (Bergsbaken *et al*., 2009). But inhibition of caspase-1 by 50 µM of zYVAD had no effect on the *M. tuberculosis*-induced necrosis (Fig. 2D). Taken together, we concluded that NLRP3 activation by ESAT-6 played a key role in mediating macrophage necrotic death, independent of caspase-1, by *M. tuberculosis*.

### The role of Zmp1 on NLRP3 functions during *M. tuberculosis* infection

**Figure 3:**
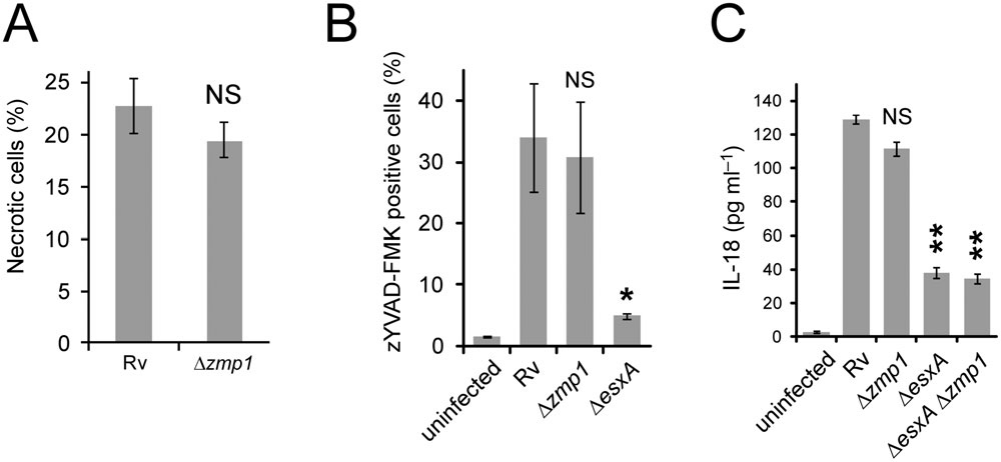
The role of Zmp1 on NLRP3 functions during *M. tuberculosis* infections. Differentiated THP-1 macrophages were infected with the indicated strains of *M. tuberculosis* H37Rv for 2 days. A. The percentages of infected THP-1 macrophages that became necrotic were determined by fluorescence microscopy. B. The levels of active caspase-1 were determined by staining of active caspase-1 with zYVAD-FMK using fluorescence microscopy. C. ELISA measurement of IL-18 was performed from culture supernatants of THP-1 macrophages infected with the indicated strains. Results are summarized as means ± standard errors. **P < 0.005; *P < 0.05; NS: not significant (relative to H37Rv).

Recently, a secreted *M. tuberculosis* metalloprotease called Zmp1 is shown to inhibit the inflammasome activation by nigericin, a well-characterized NLRP3 stimulus (Master *et al*., 2008). To determine the role of Zmp1 in NLRP3 activation by *M. tuberculosis*, we constructed the Δ*zmp1* strain and found that loss of Zmp1 had no observable effect on the necrosis induced by *M. tuberculosis* in primary human macrophages (Fig. 3A) or in THP-1 macrophages (data not shown), nor the caspase-1 activation and secretions of IL-1beta in THP-1 human macrophages (Fig. 3B and C). Comparison of the double mutant Δ*esxA*Δ*zmp1* with the mutant Δ*esxA* failed to indicate a role for Zmp1 in regulating IL-18 secretion (data not shown) and necrosis (Fig. 3C). These results suggested that the mycobacterial-encoded Zmp1 played a minor role in modifying the NLRP3 activation and the induction of necrotic death by ESAT-6 during *M. tuberculosis* infection.

### ESAT-6 damages the phagosomal membrane

**Figure 4:**
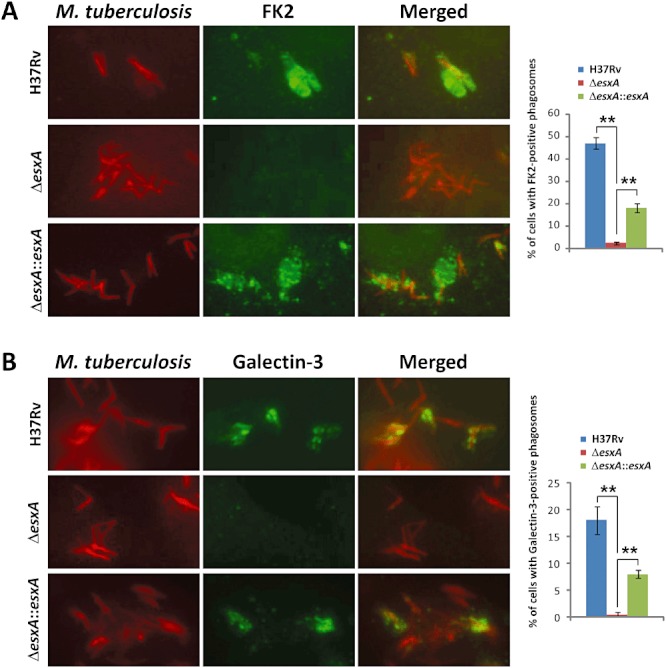
Phagosomal membrane damage by *M. tuberculosis*. A–B. ESAT-6-dependent accumulation of ubiquitinated proteins based on FK2 antibody staining (green) (A) or the phagosomal lysis marker Galectin-3 (green) (B) occurs on phagosomes containing *M. tuberculosis* (red) after 2 days post infection. Summarized results are shown in the right panel. *M. tuberculosis* is visualized by its autofluorescence upon excitation at blue wavelength and is pseudocoloured in red. Summarized results are shown in the right panel. **P < 0.001.

NLRP3 activation usually involves destabilization of phagosomal compartments (Hornung and Latz, 2010; Schroder and Tschopp, 2010). ESAT-6 is known to possess pore-forming activity, but its effect on phagosome integrity had not been determined previously. One mechanism of phagosomal destabilization involves inflicting damage on phagosomal membranes. Dupont *et al*. have recently reported that a damaged phagosomal membrane is marked by ubiquitinated proteins and galectin-3 (Dupont *et al*., 2009; Paz *et al*., 2010). We observed an ESAT-6-dependent recruitment of ubiquitinated proteins and galectin-3 onto *M. tuberculosis-*containing phagosomes (Fig. 4). These results indicated that membranes of the *M. tuberculosis-*containing phagosomes were likely damaged.

**Figure 5:**
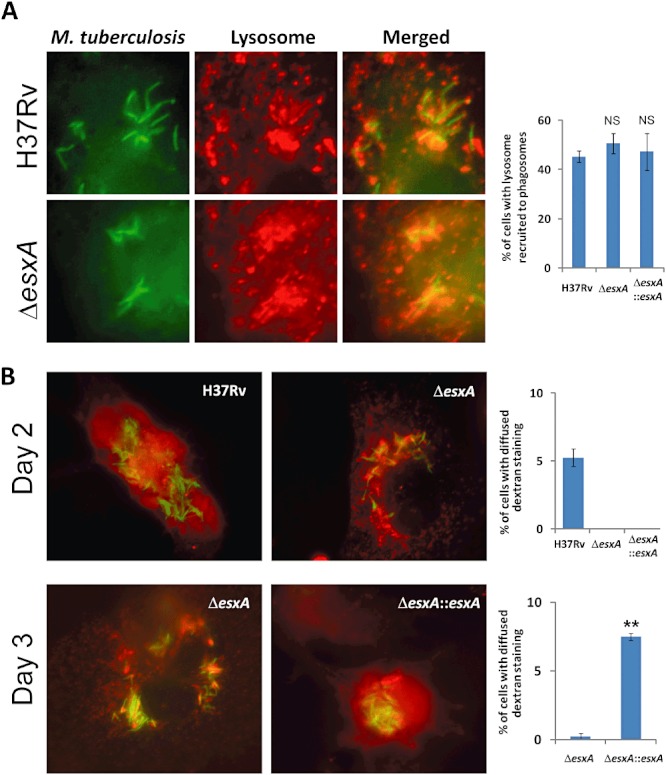
Recruitment of endolysosomes to *M. tuberculosis-*containing phagosomes and the subsequent permeabilization of endolysosomal membranes. Endolysosomal compartments of THP-1 macrophages were preloaded with 0.2 mg ml^−1^ of dextran-10 kDa (red) overnight, followed by a 2 h chase, as described in *Experimental procedures*, before infection with *M. tuberculosis*. A. Endolysosome (red) recruitment to *M. tuberculosis* (green) at 16 h post infection. The right panel shows the summarized results of the percentages of macrophages containing endolysosomal recruitment to *M. tuberculosis*, which were determined from 50 macrophages in triplicate. B. Endolysosomal permeabilization as evidenced by diffused localization of the dextran endolysosomal content marker (red) at 44 and 64 h post infection. *M. tuberculosis* was visualized by its autofluorescence and was pseudocoloured in green. At 64 h, H37Rv-infected macrophages were not scored because most of them were completely lysed, leaving no more detectable endolysosomal marker. Summarized results of the percentages of infected cells (*n* = 50 in triplicate) with diffused dextran localization are shown in the right panels. **P < 0.001; NS: not significant.

*Mycobacterium tuberculosis*-containing phagosomes in human macrophages are known to recruit markers of endosomes and lysosomes shortly after phagocytosis (van der Wel *et al*., 2007). Thus, endolysosomes likely interact with phagosomes harbouring *M. tuberculosis*, raising the possibility that endolysosomal content will be leaked into the cytosolic space because of the membrane-damaging effect of ESAT-6. We therefore assessed the fate of the endolysosomal content marker dextran-10 kDa over the course of *M. tuberculosis* infection. As expected, dextran-10 kDa was recruited to *M. tuberculosis*-containing phagosomes. This recruitment was independent of ESAT-6 (Fig. 5A). At day 2 post infection, leakage of dextran-10 kDa into the cytosol of *M. tuberculosis*-infected cells was observed. Importantly, the leakage required the presence of ESAT-6 (Fig. 5B). This result indicated that the early interaction of endolysosome with phagosome was followed by an ESAT-6-dependent permeabilization of the phagosomal compartments, which allowed leakage of the endolysosomal content into the cytosol.

### NLRP3 activation and necrosis are dependent on the ability of ESAT-6 to damage phagosomes

**Figure 6:**
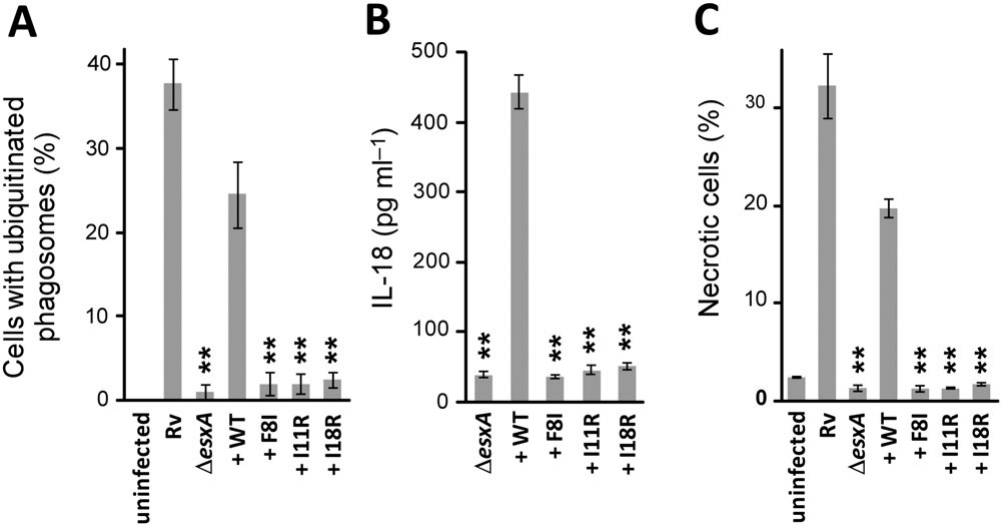
Triggering NLRP3 functions by ESAT-6 depends on the ability of ESAT-6 to cause phagosomal damage. PMA-differentiated THP-1 macrophages were infected with H37Rv, Δ*esxA*, Δ*esxA::esxA* (+ WT), Δ*esxA::esxA* (+ F8I), Δ*esxA::esxA* (+ I11R) or Δ*esxA::esxA* (+ I18R). A. Phagosomal damage of infected cells was assessed by accumulation of ubiquitinated proteins. IL-18 production (B) and necrosis (C) were examined as described in *Experimental procedures*. Results are summarized as means ± standard errors. **P < 0.005 [relative to Δ*esxA::esxA* (+ WT)].

To determine the significance of the ESAT-6-promoted phagosomal damage, we sought to generate ESAT-6 mutants that no longer cause ubiquitination of phagosomes. Recent data indicate that NLRP3 activators typically form intracellular crystals or aggregates (Hornung and Latz, 2010). Interestingly, ESAT-6, upon being overproduced in *E. coli*, forms amyloid-like insoluble aggregates (Wang *et al*., 2008). N-terminal residues in ESAT-6 critical for the formation of amyloid-like aggregates were mutated and tested. ESAT-6 point mutant F8I, I11R or I18R did accumulate ubiquitinated proteins on *M. tuberculosis-*containing phagosomes, suggesting that these ESAT-6 mutants were unable to destabilize phagosomes (Fig. 6A). Next, we examined the extent to which these mutants were defective to activate NLRP3 functions. Neither NLRP3 inflammasome activity, as assayed by the production of IL-18, nor necrosis, was upregulated by these ESAT-6 mutants during *M. tuberculosis* infection (Fig. 6B and C). The ESAT-6 mutants were also defective for endolysosomal content leakage (Fig. S1). Thus, we concluded that NLRP3 activation by ESAT-6 was dependent on the ability of ESAT-6 to damage phagosomal compartments.

### Syk kinase is required for NLRP3 functions during *M. tuberculosis* infection

**Figure 7:**
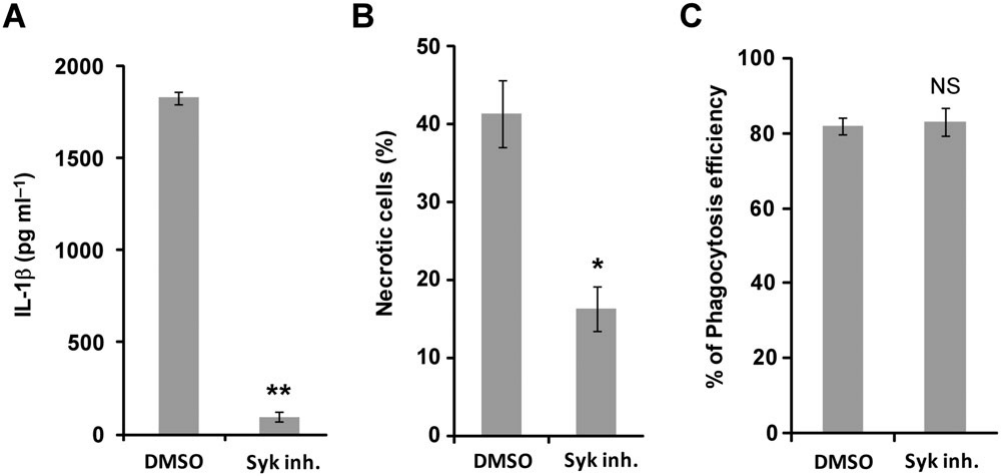
Inhibition of Syk kinase blocks IL-18 production and necrosis. PMA-differentiated THP-1 macrophages were pretreated with DMSO or 10 µM syk inhibitor. After 4 h of infection, cells were incubated in the presence of DMSO or syk inhibitor overnight. A. IL-18 levels in supernatants of THP-1 cells infected with H37Rv were assayed by ELISA. B. The percentage of necrotic cells infected with H37Rv was determined from propidium iodide staining under fluorescence microscopy. **P < 0.001; *P < 0.01; NS: not significant (relative to DMSO control).

The previous result showed that ESAT-6 residues critical for forming amyloid-like aggregate were essential for the phagosome-damaging effect of ESAT-6. Amyloid-beta is known to stimulate IL-1beta production in THP-1 monocytes, and this innate response towards amyloid-beta is dependent on Syk kinase activity (Combs *et al*., 2001). In addition, Syk kinase has recently been shown to mediate NLRP3 activation in response to fungal or malarial infection (Gross *et al*., 2009; Shio *et al*., 2009). We therefore tested whether the ESAT-6-NLRP3-dependent responses in THP-1 macrophages infected with *M. tuberculosis* were mediated by Syk kinase activity. Indeed, treatment of macrophages with a Syk inhibitor potently prevented the *M. tuberculosis*-induced necrosis (Fig. 7A), as well as the IL-1beta production (Fig. 7B). Syk activity is required for phagocytosis. To rule out the possibility that inhibition of Syk activity prevented NLRP3 activation simply by blocking phagocytosis of *M. tuberculosis* by macrophages, the effect of Syk inhibition on phagocytosis efficiency was determined. We found that treatment of the Syk inhibitor had no effect on the ability of macrophages to take up *M. tuberculosis* (Fig. 7C). Thus, we uncovered a key requirement for Syk kinase activity in NLRP3-dependent necrotic induction and IL-1beta production promoted by *M. tuberculosis*. These findings might suggest a mechanism of NLRP3 activation.

We then determined the extent to which ESAT-6 mediated Syk activation. The NLRP3 activator haemozoin from *Plasmodium* stimulates Syk phosphorylation during early interactions with macrophages (Shio *et al*., 2009). Syk phosphorylation is a key step for Syk activation. Using a monoclonal antibody specific for phosphorylated Syk, we occasionally observed the presence of phosphorylated Syk around *M. tuberculosis*. However, Syk phosphorylation occurred at the same rate for *M. tuberculosis*Δ*esxA* (data not shown), implying that Syk was activated by a *M. tuberculosis* factor independent of ESAT-6. Finally, we examined the possibility that phagosomal damage was dependent on Syk signalling. Ubiquitination stimulated by *M. tuberculosis*, a phagosomal-damage event, was unaffected by the Syk inhibition (data not shown). Thus, Syk signalling and phagosome damage were two necessary but independent factors that mediate NLRP3 activation for necrotic death.

## Discussion

In this study, we explored the mechanism through which *M. tuberculosis* utilizes ESAT-6 to trigger necrotic death in human macrophages. Our results suggested a critical role for the NLRP3 inflammasome, which has been shown to be activated by ESAT-6, in promoting the necrotic death triggered by *M. tuberculosis* in human macrophages. The phagosomal damaging effect of ESAT-6 and the host Syk kinase activity were further implicated as two signalling events that relay innate recognition of ESAT-6 to NLRP3 activation.

The NLRP3 inflammasome is emerging as a key regulator for multiple examples of bacterial-induced necrotic death. However, how NLRP3 contributes to necrosis remains poorly understood. In the case of *Salmonella* and *Listeria* infections, necrosis induction requires the caspase-1-mediated inflammasome of NLRP3, although the cascade of events downstream of caspase-1 that are responsible for cell death remains unknown (Bergsbaken *et al*., 2009). We have observed that the NLRP3-dependent necrosis promoted by *M. tuberculosis* is independent of caspase-1 (Fig. 2D). Caspase-1-independent necrosis mediated by NLRP3 has also been reported. In macrophages infected with *Shigella* and *Staphylococcus*, the ASC adaptor that links caspase-1 to NLRP3 is required for inducing necrosis, whereas caspase-1 is not required (Willingham *et al*., 2007; Craven *et al*., 2009). It remains to be elucidated whether ASC facilitates necrosis independent of caspase-1 and, if so, how it does so. In addition, a better understanding of the role ASC plays in *M. tuberculosis*-induced necrosis is needed.

The NLRP3 inflammasome can be activated by pore-forming inducers, such as bacterial pore-forming toxins and ATP, or by phagolysosomal destabilizers, such as cholesterol, alum and amyloid-beta. All the cases of NLRP3-dependent bacterial-triggered necrosis reported so far required a pore-forming activity (Willingham *et al*., 2007; Craven *et al*., 2009; Toma *et al*., 2010), whereas the role of phagosomal damage in bacterial-triggered necrosis is less clear. Pore-forming activity of ESAT-6 has been demonstrated (Hsu *et al*., 2003; Derrick and Morris, 2007; Smith *et al*., 2008), but a damaging effect on phagosomes by ESAT-6 has not been reported before. Results based on several independent assays led us to conclude that phagosomes containing *M. tuberculosis* were damaged: first, lysosomal content marker dextran-10 kDa was initially recruited to the phagosomes, which was then followed by diffusion into the cytosol; and second, ubiquitinated proteins and galectin-3, both recently linked to bacterial-damaged phagosomal membrane, were markedly recruited to *M. tuberculosis* in an ESAT-6-dependent fashion. Translocation of *M. tuberculosis* from phagosome to cytosol has been shown to be mediated by the ESX1 secretion system. Determining whether and, if so, how this cytosolic translocation is linked to the ESAT-6-dependent phagosomal damage requires further investigation.

Using mutants of ESAT-6 that lost their abilities to accumulate ubiquitinated aggregates as a marker for destabilized phagosomes, we showed that the phagosomal destabilizing effect of ESAT-6 was required to activate NLRP3 functions such as necrosis and production of inflammatory cytokines. Thus, our findings underscored the significance of the ESAT-6-induced phagosomal destabilization for NLRP3 activation.

The permeability of *M. tuberculosis*-containing phagosomes has previously been suggested (van der Wel *et al*., 2007; Smith *et al*., 2008). Lysosomes were recruited to the *M. tuberculosis-*containing phagosomes (Fig. 5A) and leakage of lysosomal contents could be detected in an ESAT-6-dependent fashion (Fig. 5B). Compartmental content of the phagolysosomes, including factors derived from the host macrophages or the bacteria, could reach cytosolic spaces. Cytosolic release of lysosomal cathepsin B has been suggested as partially mediating NLRP3 activation (Hornung and Latz, 2010), although cathepsin B activity was also shown to promote lysosomal rupture under different conditions (Fujisawa *et al*., 2007). However, we found no effect of the inhibition of cathepsin B by CA-074Me on *M. tuberculosis*-induced necrosis (data not shown). Lysosomal lipases, such as sphingomyelinase and phospholipase A2, have been known to mediate lysosome-mediated cell death (Guicciardi *et al*., 2004). Nevertheless, chemical inhibitions against these lipases by chlorapmazine revealed no observable protective effect against ESAT-6-triggered necrosis during *M. tuberculosis* infection (data not shown). Thus, we were unable to reveal a major role of lysosomal leakage in necrosis induction. We still do not know whether lysosomes were damaged by an ESAT-6-dependent mechanism. Lysosomal leakage is likely caused by the damaged phagosomes after interaction with lysosomes. As ESAT-6 appeared to be more efficient to promote phagosomal damage (Fig. 4) than to promote lysosomal leakage (Fig. 5), we considered that NLRP3 activation is more likely mediated by a phagosomal content, rather than a lysosomal content. Bacterial factors including muramyl dipeptide (MDP) and RNA have been reported to activate NLRP3 (Martinon *et al*., 2004; Kanneganti *et al*., 2006). Indeed, cytosolic MDP recognition by the Nod2 sensor is dependent on the ESX-1 secretion system of *M. tuberculosis* (Pandey *et al*., 2009), raising an unverified possibility that delivery of MDP or other mycobacterial-derived factors from a phagosomal environment through ESAT-6 could activate the cytosolic NLRP3 inflammasome.

Studies on the regulation of NLRP3 activation have implicated reactive oxygen species (ROS), potassium ion efflux and Syk activity as signalling mediators for NLRP3 activation (Franchi *et al*., 2010, Schroder and Tschopp, 2010). However, a role for ROS in NLRP3 activation remains controversial (van Bruggen *et al*., 2010; van de Veerdonk *et al*., 2010). In our case of *M. tuberculosis* infection of human macrophages, treatment with the ROS-scavenger N-acetyl-lysines conferred no protection for infected macrophages (data not shown). Potassium ion efflux has recently been implicated in the induction of IL-1beta by *M. tuberculosis* (Kurenuma *et al*., 2009). Nevertheless, the presence of extracellular 10–40 mM KCl, which prevents efflux of the potassium ion, had no effect on the *M. tuberculosis*-induced death in our hands (data not shown). Instead, we reported a key role for Syk in supporting NLRP3 functions during *M. tuberculosis* infection. It is interesting to note that a major role for Syk in mediating NLRP3-dependent responses has not been reported for bacterial infections. Only a minor role for Syk has been reported in the case of IL-1beta production stimulated by *Chlamydia* infection (Abdul-Sater *et al*., 2010), whereas Syk played a major role in NLRP3 activation during fungal or malarial infections. How Syk mediates NLRP3 activation by *M. tuberculosis* without the involvement of ROS and potassium efflux remains unclear. Syk may activate NLRP3 through its reported ability to interact directly with ASC, an adaptor for the assembly of the caspase-1 inflammasome (Shio *et al*., 2009). Syk has also been implicated in lysosome-dependent cell death after B cell receptor engagement (He *et al*., 2005). Thus, an alternate scenario involves Syk affecting the phagosomal damaging ability of ESAT-6 and hence NLRP3 activation. However, inhibition of Syk activity had no impact on the effects of ESAT-6 on phagosomes – such as Gal-3 recruitment, ubiquitinated protein accumulation and lysosome leakage (Fig. 4 and data not shown). This observation indicated that Syk may sense the ESAT-6-induced phagosomal damage or its related events. Alternatively, an ESAT-6-triggered event unlinked to destabilized phagosomes could be responsible. Regarding how *M. tuberculosis* activates Syk, the responsible *M. tuberculosis* factor remains unknown, although we have ruled out a role for ESAT-6. A recent study identifies Mincle as the long-sought receptor for mycobacterial trehalose dimycolate (Ishikawa *et al*., 2009). Interestingly, the signal transduction downstream of Mincle involves Syk kinase for its signal transduction (Werninghaus *et al*., 2009). Additional studies are needed to determine whether trehalose dimycolate contributes to Syk activation.

Master *et al*. have recently reported that BCG strain is capable of preventing inflammasome activation induced by nigericin (Master *et al*., 2008). This inhibition is likely mediated by Zmp1, as BCG strain lacking Zmp1 induced significantly more IL-1beta. Their results are inconsistent with our present result, which could be due to the different choices of macrophages. Another possible explanation is the difference in the strain background. Master *et al*. used BCG, which does not express the ESX-1 secretion system. Without ESX-1, the effect of Zmp1, we reasoned, could be more pronounced. We verified this idea by deleting *zmp1* in the H37Rv lacking *esxA*. This double mutant, however, induced similar IL-18 level when compared with H37Rv Δ*esxA*. Our result indicated that Zmp1 did not modify the ESX-1-promoted NLRP3 activation. It is possible that BCG strain lacks a Rv-specific factor that can prevent the effect of Zmp1.

It is tempting to speculate based on our present results that the NLRP3-activating effect of ESAT-6 is due to the amyloid-like structure formation by ESAT-6. We found that activation of NLRP3-dependent functions by *M. tuberculosis* ESAT-6 was dependent on ESAT-6 residues that were specifically required for forming amyloid-like structures in *E. coli*, as well as on Syk kinase activity, which is critical for the amyloid-beta-stimulated IL-1beta production in monocytes and microglia. It is possible that the hypothetical amyloid structure is linked to the known pore-forming activity of ESAT-6. However, we were unable to detect any amyloid-like aggregate in infected macrophages using anti-amyloid antibody (A11) and amyloid-specific stain thioflavine T. It is possible that our methods were not sensitive enough to detect endogenous level of amyloid-like structure, which could be sensed by macrophages at a very low concentration. Thus, the concept of amyloid-like aggregation of ESAT-6 remains to be demonstrated.

In summary, necrotic death in *M. tuberculosis*-infected macrophages is governed by the NLRP3 inflammasome. We further implicated phagosomal permeabilization and Syk activity as two of the mechanisms of NLRP3 activation. Because macrophage necrotic death is central to tuberculosis pathogenesis, targeting NLRP3 to block macrophage necrotic death may represent an attractive therapeutic approach. However, a drawback of this approach is that the inflammasome activity of NLRP3, which has been shown to play a key protective role in host defence against *M. tuberculosis* infections, would also be blocked. This possibility is supported by a preliminary study, in which we found that a long-term treatment of glyburide to inhibit NLRP3 did not extend the survival of *M. tuberculosis*-infected mice (data not shown). A similar result based on *M. tuberculosis* infections in NLRP3-deficient mice has also recently been reported (McElvania Tekippe *et al*., 2010). We foresee that a key direction for future research is identifying which the NLRP3-regulated components play a critical role in mediating necrotic death, but not in other inflammasome activities.

## Experimental procedures

### Cell culture

The human monocytic cell line THP-1 was obtained from ATCC (Manassas, VA, USA) and cultivated in RPMI 1640 (Cat. 11875, Gibco, Invitrogen, Carlsbad, CA, USA) containing 10% heat-inactivated Hyclone fetal bovine serum (Thermo Scientific, Waltham, MA, USA) and 10 mM Hepes. Cells were grown at 37°C in a humidified chamber with 5% CO_2_. THP-1 monocytes/macrophages were prepared by differentiating the cells in the presence of 100 nM phorbol myristate acetate (PMA) for 3 days in 48-well tissue culture-treated Costar plates (Corning, Corning, NY, USA), followed by 1 day of rest in culture medium without PMA. CD14^+^ monocytes were isolated from human PBMC by using a positive selection CD14 column according to the manufacturer's protocol (Miltenyi Biotech, Auburn, CA, USA). Selected monocytes were allowed to differentiate into macrophages in a 48-well Costar plate for 7 days before infection. Cell densities were typically adjusted to 1 × 10^6^ cells ml^−1^ and 0.2 ml or 0.4 ml of cells was dispensed into each well of a 48-well plate or 24-well plate respectively.

### Construction and complementation of Δ*esxA* mutant

The Δ*esxA* (Rv3875) mutant was constructed by homologous recombination using specialized transduction of a phAE159 phage that carried the *res-sacB-hyg-res* cassette containing the *sacB* and hygromycin resistance genes flanked by upstream and downstream DNA regions of *esxA* (T. Hsu and W. R. Jacobs, Jr, unpubl. results), as previously described (Bardarov *et al*., 2002). To complement Δ*esxA*, Δ*esxA* was transformed with the integrative plasmid pMV361::*esxA*, which was constructed by PCR directional cloning of the *esxA* gene into the restriction sites of pMV361-*kan^R^* to generate the complemented strain Δ*esxA attB_L5_*::pMV361::*esxA* (Δ*esxA*::*esxA*). Based on a Western blot analysis using a monoclonal antibody against ESAT-6 (HYB 076–08) (Thermo Scientific), the complementated strain expressed a lower level of ESAT-6 in comparison with the wild-type H37Rv strain when grown in 7H9 medium, whereas the Δ*esxA* mutant had no detectable ESAT-6 expression under the same condition (Fig. S2). The ESAT-6 point mutations F8I, I11R, and I18R were introduced individually into pMV361::*esxA* using Quikchange site-directed mutagenesis kit (Stratagene, La Jolla, CA, USA). Mutation at residue 8 of ESAT-6 has previously been shown to have no effect on the secretion of ESAT-6 and CFP-10, but have a severe defect in *in vivo* growth in lungs of mice (Brodin *et al*., 2005).

### Reagents

The following concentrations of inhibitors were used to treat monocytes/macrophages: 50 µM glyburide, 50 µM glipizide (Sigma), 10 µM Syk inhibitor IV (Calbiochem, EMD Chemicals, Gibbstown, NY, USA), benzyloxycarbonyl-Tyr-Val-Ala-Asp-fluoromethylketone (zYVAD; Calbiochem). Equivalent volumes of DMSO were used as vehicle control.

### Infection conditions

*Mycobacterium tuberculosis* was grown on Middlebrook 7H9 medium (Difco, Becton Dickinson, Franklin Lakes, NJ, USA) with complete supplements [10% OADC (BBL, Becton Dickinson), 0.5% glycerol and 0.05% Tween-80]. 30 µg ml^−1^ of kanamycin was added to the medium for complemented strains. Mycobacterial cultures were grown to logarithmic phase or an OD_600_ of between 0.4 and 0.75. Before infection, bacteria were pelleted in a 2 ml tube with attached loop cap (Cat. 72.694.106, SARSTEDT, Newton, NC, USA), resuspended in 1 ml of PBS (without Tween-80) in a 15 ml conical tube, and sonicated twice for 10 s (80 output and 100% duty cycle) in a water-jacketed cup horn connected to a Branson Sonifier 250 (Branson, Danbury, CT, USA). The OD_600_ of each strain was then measured. To achieve a multiplicity of infection of 5 or 10, sonicated bacteria were diluted in pre-warmed tissue culture medium to 5 × 10^6^ or 10 × 10^6^ CFU ml^−1^, respectively, assuming that 1 OD_600_ is equal to about 3 × 10^8^ CFU ml^−1^. For inhibitor studies, monocytes/macrophages were treated for at least 1 h before infection with chemical inhibitors or DMSO control that was diluted in complete tissue culture medium. For infection of human monocytes/macrophages, 10% normal human serum (Cat. 100–110, Gemini Bio-Products, West Sacramento, CA, USA) was used instead to facilitate mycobacterial binding to host cells. 0.2 ml or 0.4 ml of diluted bacteria was then used to replace the tissue culture medium for the adherent monocytes/macrophages in each well of a 48-well plate (Cat. 3548, Corning) or a 24-well plate (Cat. 3524, Corning) respectively. After 4 h incubation at 37°C, infected cells were washed twice gently with room temperature PBS and incubated for 1–3 days in fresh complete tissue culture medium and, when appropriate, protease inhibitors or DMSO control was included.

### NLRP3 knockdown by RNAi

THP-1 was first treated with 100 nM PMA overnight in a 48-well plate. On the next day, 100 nM siRNA against NLRP3 (ON-TARGETplus SMARTpool L-017367–00-0005: GGAUCAAACUACUCUGUGA, UGCAAGAUCUCUCAGCAAA, GAAGUGGGGUUCAGAUAAU, and GCAAGACCAAGACGUGUGA; Dharmacon, Thermo Scientific, USA) or non-targeting RNAi control (ON-TARGETplus Non-targeting Pool D-001810–10-05; Dharmacon, Thermo Scientific, USA) preincubated with Transfection Reagent 2 (Dharmacon) was used to treat the THP-1 for 2 days in the presence of 100 nM PMA. After 2 days of siRNA treatment (or a total 3 days of PMA treatment), THP-1 was rested for 1 day in culture medium without PMA, before being infected with *M. tuberculosis*.

### Determination of siRNA knockdown efficiency of NLRP3

Total RNAs were extracted from THP-1 infected with H37Rv for 21 or 41 h using RNeasy Protect Cell Mini Kit (Qiagen, USA). RNA concentrations were measured by Nanodrop (Thermo Scientific, USA). 200 ng of RNA was primed with random hexamers and reverse-transcripted into cDNAs using SuperScript III First-Strand Systhesis System (Invitrogen, USA). The cDNA was amplified by real-time PCR on ABI PRISM 7900HT sequence detector system (Applied Biosystems, Foster City, CA, USA) in triplicate using Platinum SYBR Green qPCR SuperMix-UDG (Invitrogen, USA) with primers specific to NLRP3 and 18S as described (Rajamaki *et al*., 2010). Relative NLRP3 expressions were normalized to 18S rRNA as an endogenous control for total cDNA input and were calculated based on a comparative Ct method (Livak and Schmittgen, 2001).

### Cytokine measurements

Culture supernatants were filtered in a spin column with a 0.22 µm filter (Cat. 8169, Corning) before being taken out of a BSL3 laboratory, and then assayed by enzyme-linked immunoabsorbent assay (ELISA) kits to measure human IL-1, and human IL-6 (Biosource, Invitrogen), and human IL-18 (MBL international, Woburn, MA, USA).

### Necrotic cell and active caspase-1 staining

Infected cells were stained by 2.5 µg ml^−1^ of propidium iodide (Sigma) in RPMI without serum for 10 min at RT. Macrophages were stained for active caspase-1 using FAM-YVAD-FMK (Immunochemistry Technologies, Bloomington, MN, USA) according to the manufacturer's protocol for the last hour of infection. Stained cells were washed twice with RPMI without serum, and then fixed in 2% paraformaldehyde (Sigma) in RPMI for 1 h at 37°C. After fixation, infected cells were removed from BSL3 facilities and analysed using a 10 × objective mounted on an Olympics inverted microscope with a 100-W Hg light source. Images were captured by an ORCA-II Hamamatsu camera (Hamamatsu, Bridgewater, NJ, USA) controlled by MetaMorph ver. 3.5 (Molecular Devices, Downington, PA, USA). Brightness and contrast were adjusted linearly in Photoshop (Adobe). Percentage of necrotic cells was defined as the number of propidium iodide-positive cells relative to the total number of cells. Data were displayed as the mean percentage obtained from three different fields; at least 1000 cells were examined from each field.

### Phagocytosis efficiency of M. tuberculosis by THP-1 macrophages

The efficiency of THP-1 macrophages to take up *M. tuberculosis* after 4 h of infection was performed as described (Vilcheze *et al*., 2010). Briefly, infected cells were fixed in 2% paraformaldehyde containing culture medium for 1 h at 37°C. After blocking in 10% goat serum for 10 min at room temperature, extracellular bacteria were stained by a *Mycobacterium*-specific rabbit polyclonal antibody (Cygnus Technologies, Southport, NC, USA). Extracellular bacteria were visualized by anti-rabbit secondary antibody conjugated to Alexa Fluor 488 (Invitrogen), whereas total bacteria were visualized by the autofluorescence of *Mycobacterium* as indicated below. Infected cells typically had 5–10 bacteria. Cells were considered positive for phagocytosis when they had less than two extracellular bacteria. At least 100 infected cells were counted in triplicate under a fluorescent microscopy through a 100× objective.

### Immunofluorescence staining of phagosomal damages

THP-1 human macrophages were grown on coverslips, infected and fixed as indicated above. Coverslips were permeabilized with brief treatment of cold methanol, followed by incubating in blocking buffer (PBS with 10% goat serum). Phagosomal membrane damage was stained by antibodies to ubiquitinated proteins (FK2) (Enzo Life Science) and galectin-3 (BD Pharmingen) diluted in blocking buffer. The antibodies were visualized by secondary antibodies conjugated to Alexa Fluor 488 or 594 (Invitrogen). *M. tuberculosis* was visualized by their blue autofluorescence as indicated below. An infected cell was considered positive when at least one of its *M. tuberculosis-*containing phagosomes were stained positive for the phagosomal damage marker. The percentages of infected cells having at least one *M. tuberculosis-*containing phagosome that was positive for ubiquitinated protein or galectin-3 were determined from 50 cells in quintuplicate.

### Endolysosome trafficking and permeability assays

Endolysosomal compartments were labelled as described (Huynh *et al*., 2007). 0.2 mg ml^−1^ dextran-10 kDa conjugated to Texas Red (fixable) (Invitrogen) was loaded onto PMA-differentiated THP-1 overnight, followed by a 2 h chase. The percentages of cells containing *M. tuberculosis* that had recruited endolysosomes were determined from 50 infected cells from four coverslips. The percentages of infected cells having diffused dextran throughout the cells were scored from 50 infected cells in quadruplicate.

### Direct visualization of *M. tuberculosis* associated with macrophages

We serendipitously discovered that *M. tuberculosis* grown either in liquid culture or inside macrophages exhibited blue autofluorescence upon excitation with 390–410 nm. To visualize *M. tuberculosis* associated with macrophages, THP-1 macrophages grown on coverglass and infected with *M. tuberculosis* were fixed in 2% paraformaldehyde in RPMI. Coverglass was then mounted on a glass slide using Antifade reagent (Invitrogen). *M. tuberculosis* was imaged using the standard filter for 4′,6-diamidino-2-phenylinodole (DAPI) nucleic acid dye on an inverted fluorescent microscopy (Nikon Ti Eclipse system, Nikon, USA) equipped with a CCD-cool camera (Coolsnap HQ, Photometrics) through a 100 × objective. The blue autofluorescence, with a half-live of about 70 s, was stable enough for the imaging purpose. Green or red fluorescence was unaffected by the blue autofluorescence, permitting simultaneous monitoring of other host/bacterial factors as described above. In addition, the mature phagolysosomal environment was found to have no effect of the blue autofluorescence.

### Statistical test

Results were tested statistically by unpaired two-tailed Student's *t-*test using Excel (Microsoft).

## Notes

Fig. S1 Lysosomal leakage by ESAT-6 depends onthe ability of ESAT-6 to cause phagosomal damage.PMA-differentiated THP-1 macrophages were infected with H37Rv,Δ*esxA*, Δ*esxA::esxA* (+WT),Δ*esxA::esxA* (+F8I), Δ*esxA::esxA* (+I11R)or Δ*esxA::esxA* (+I18R). Percentages of infectedmacrophages exhibited diffused lysosomal staining were examinedfrom 100 cells (*n* = 6). Results are summarized as means± standard errors. ***P* < 0.005 [relative toΔ*esxA::esxA* (+WT)].

Fig. S2 Expression levels of ESAT-6.PMA-differentiated THP-1 macrophages were infected with H37Rv,Δ*esxA*, Δ*esxA::esxA* (+WT) were grown in7H9. 10 ml of cultures were pelleted and resuspended in 100µl of extraction buffer (1 M Tris, pH 7.6; 0.5 M EDTA; 10%SDS). Resuspended pellets were added with 100 µl of glass bead (106 µm, Sigma) and then vortexed at top speed for 5min. Next, whole cell lysates were added with 6 × SDS-PAGE sample buffer (without the bromophenol dye) so that the final concentration was 1×. Samples were incubated in a 100°Cdry bath for 5 min. Protein samples were safe at this point to betaken out from Biohazard Level 3 facility. Protein concentrations were measured by BCA assay (Pierce, Thermo Scientific) after pretreating the samples with Compat-Able Protein Assay Preparation Reagent (Pierce). 65 µg of whole cell lysates were analysedon a 10% SDS-PAGE and immunoblotted using antibodies against Hsp65 (Abcam, Cambridge, MA: ab20519) as a loading control and against ESAT-6 (Thermo Scientific Pierce Antibodies: HYB076–08-02).
