# Supplementary figures and images for: Critical role for NLRP3 in necrotic death triggered by Mycobacterium tuberculosis
Source: Cell Microbiol. 2011 Mar 11;13(9):1371–84. doi: 10.1111/j.1462-5822.2011.01625.x (PMC3257557; doi:10.1111/j.1462-5822.2011.01625.x)

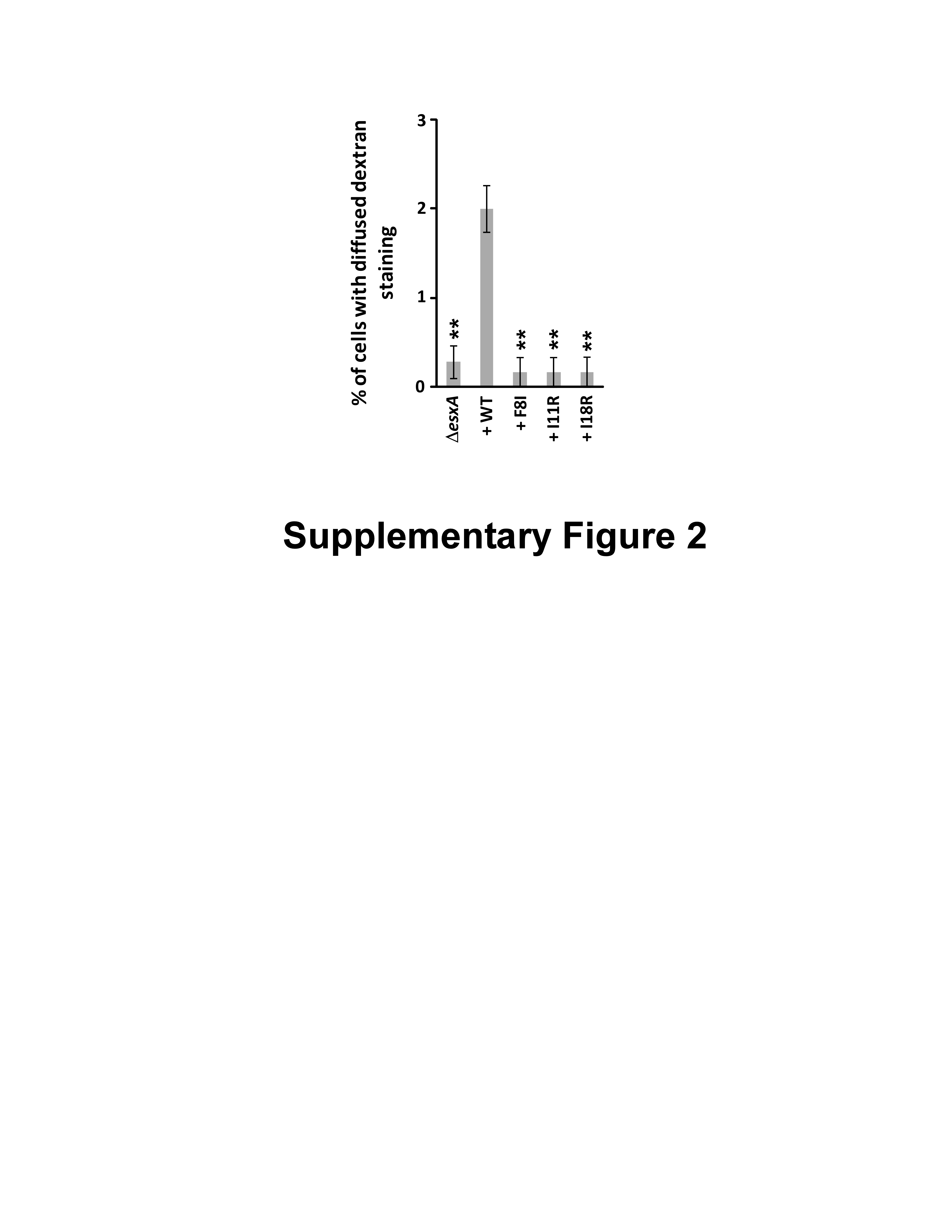

Supplement: Fig. S1 — Lysosomal leakage by ESAT-6 depends onthe ability of ESAT-6 to cause phagosomal damage.PMA-differentiated THP-1 macrophages were infected with H37Rv,ΔesxA, ΔesxA::esxA (+WT),ΔesxA::esxA (+F8I), ΔesxA::esxA (+I11R)or ΔesxA::esxA (+I18R). Percentages of infectedmacrophages exhibited diffused lysosomal staining were examinedfrom 100 cells (n = 6). Results are summarized as means± standard errors. **P < 0.005 [relative toΔesxA::esxA (+WT)]. [file cmi0013-1371-SD1.tif]

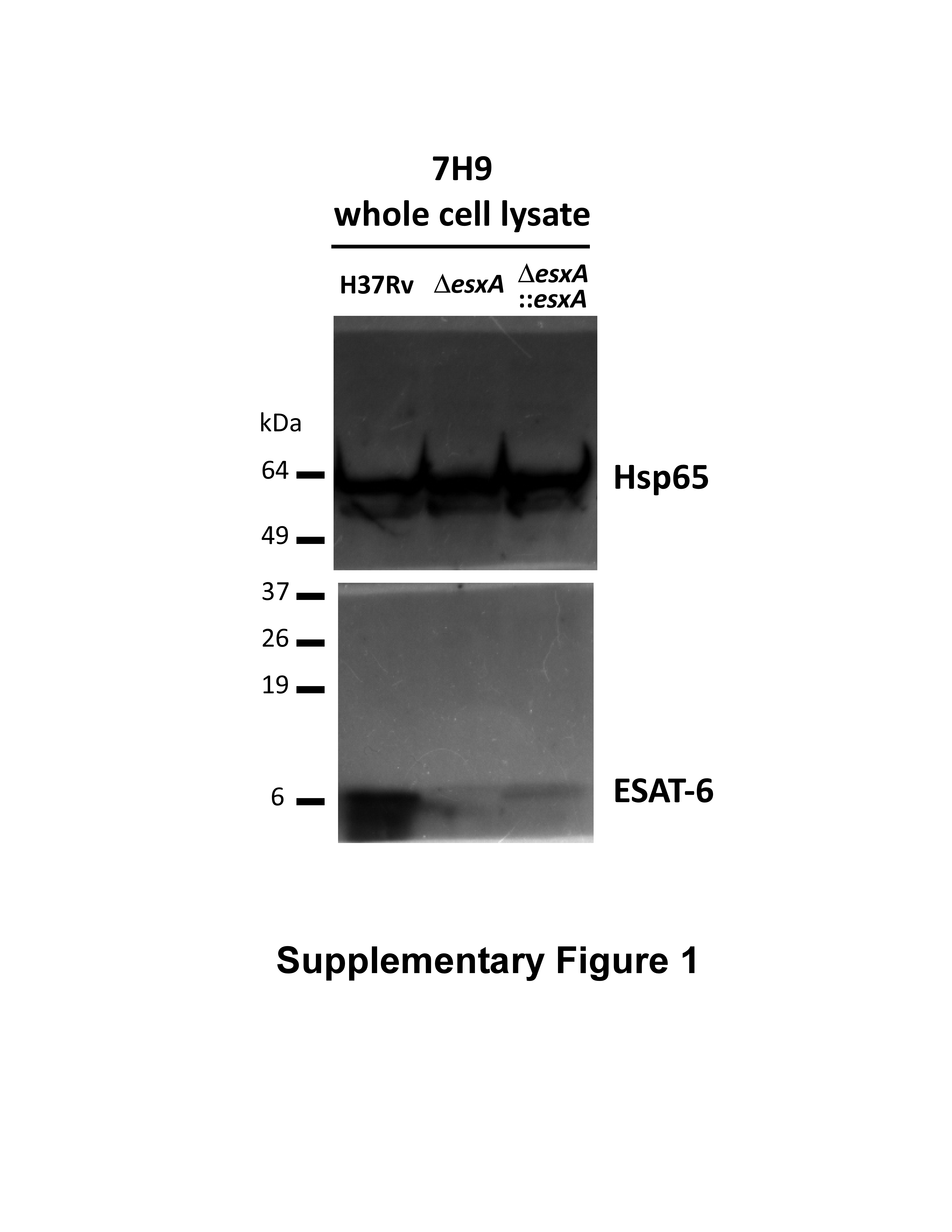

Supplement: Fig. S2 — Expression levels of ESAT-6.PMA-differentiated THP-1 macrophages were infected with H37Rv,ΔesxA, ΔesxA::esxA (+WT) were grown in7H9. 10 ml of cultures were pelleted and resuspended in 100µl of extraction buffer (1 M Tris, pH 7.6; 0.5 M EDTA; 10%SDS). Resuspended pellets were added with 100 µl of glass bead (106 µm, Sigma) and then vortexed at top speed for 5min. Next, whole cell lysates were added with 6 × SDS-PAGE sample buffer (without the bromophenol dye) so that the final concentration was 1×. Samples were incubated in a 100°Cdry bath for 5 min. Protein samples were safe at this point to betaken out from Biohazard Level 3 facility. Protein concentrations were measured by BCA assay (Pierce, Thermo Scientific) after pretreating the samples with Compat-Able Protein Assay Preparation Reagent (Pierce). 65 µg of whole cell lysates were analysedon a 10% SDS-PAGE and immunoblotted using antibodies against Hsp65 (Abcam, Cambridge, MA: ab20519) as a loading control and against ESAT-6 (Thermo Scientific Pierce Antibodies: HYB076–08-02). [file cmi0013-1371-SD2.tif]
